# Editorial Department

**Published:** 1874-05-15

**Authors:** 


### Editorial Department.

ILLINOIS STATE MEDICAL SOCIETY.—The Committee of Arrangements have not been able to secure any reliable arrangement with the several Railroad Companies for reduction of fare, either to or from the place of meeting. Neither is it practicable to secure any arrangements with the leading hotels for reduction of the ordinary rates of board for members attending the meetings of the Society. All propositions looking to this end involve the condition that the Committee pledge the stopping of a certain *number* at a given house. The leading first-class hotels, the Grand Pacific, the Tremont, and the Sherman, charge $4.50 per day; the

Palmer, from $3.00 to $5.00 per day, according to room. Among the more moderate-priced houses, the Commercial, corner of Lake and Dearborn streets, $2.50 per day, is good; also, the Briggs, corner of Randolph and Wells streets; the Matteson, corner of Wabash ave. and Quincy street, and the Clifton, corner of Wabash ave. and Monroe street. Members from other parts of the State may be assured that they will meet a cordial reception from the profession in the city.

The Sessions of the Society will be held in the Lecture-room of the Academy of Science, 253 Wabash avenue, near Van Buren street.

Plagiarisms.—The criticism of "Curette," in The Examiner of April 15th, has called out a reply, covering nearly twenty pages of manuscript, in which the writer attempts to show, what no one disputes, namely, that Buckle, in his History of Civilization, uses a great variety of facts that are common property for all writers, and quotes from a great many authors, which he fully acknowledges in a full list, and in marginal notes. But all this does not touch the question raised by "Curette," in his letter to The Examiner. That question was, simply, whether the address on "Organic Reform," in the Transactions of the Illinois State Medical Society, contained *verbatim* quotations to the extent of whole paragraphs, from Buckle's History, without any credit or acknowledgment therefor? If so, it constitutes what is generally called *Plagiarism*. But as the twenty pages of manuscript sent to us, do not touch this question in any way, we can hardly afford the space to publish it.

---

"Ably Edited."—We observe that the *New York Medical Journal*, in its advertisements now appearing in the newspapers, makes a great point of recommending itself to the profession by stating that it is *ably edited* by Drs. Lusk and Hunter. We agree with Drs. Lusk and Hunter, that they are able editors, but it is not generally considered necessary to tell the world what the world ought to know without blasts from one's own trumpet.—*London Medical Press and Circular.*

